# Adding resolution and dimensionality to comparative genomics: moving from reference genomes to clade genomics

**DOI:** 10.1186/s13059-018-1500-7

**Published:** 2018-08-14

**Authors:** Jeffrey Rogers

**Affiliations:** 1 Human Genome Sequencing Center, Baylor College of Medicine, Houston, TX 77030 USA; 2 Department of Molecular and Human Genetics, Baylor College of Medicine, Houston, TX 77030 USA

## Abstract

The main goal and promise of comparative genomics has been to create a comprehensive catalog of genomic information and function across the phenomenal diversity of living systems. A recent study has demonstrated the evolutionary insights possible by generating high-quality whole-genome assemblies from multiple species of a clade.

## Making impossible things feasible

The first whole-genome reference assemblies were available for only the highest priority species (humans, mice, and *Drosophila*). Happily, advances in technologies for DNA sequencing and computational analysis have made it practical to generate new high-quality genome assemblies at an accelerating pace. Excellent genomic resources are now publicly available for hundreds of plant, invertebrate, and vertebrate species, to say nothing of the remarkable databases of microbial diversity.

Nevertheless, cost remains a constraint and as a result it has been commonplace for research communities to collectively choose one key species (or a small number of species) from a major clade for thorough analysis. This leaves investigators who are working on other closely related species to ‘borrow’ information from the selected keystone representative of a given taxonomic group. Fortunately, the continuing reductions in sequencing costs, and the development of new tools that generate more robust assemblies, are progressively removing this barrier. It is increasingly feasible to generate high-quality, highly contiguous whole-genome assemblies not only for individual representatives of clades but also in parallel for multiple species within a group. This increasing granularity of comparative genomic information provides new opportunities for evolutionary analysis. The increased resolution of information facilitates the ability of investigators to estimate the timing of genomic innovations and to measure variation in rates of genomic evolution through time and across lineages. This is not possible on a sparsely annotated phylogenetic tree. These improved assemblies (with fewer gaps) can reveal patterns of evolutionary change that previously could not be discerned. For example, recent efforts have added the dimensions of segmental duplications, structural variants, and even haplotype phasing to the traditional genomic data sets of single, unphased nucleotide differences and small insertion/deletions [1].

## A new illustration from murid rodents

A valuable and informative example of this trend toward finer-scale phylogenetic resolution has recently been published by Thybert and colleagues [2]. In this study, the authors sequenced, assembled, and compared the genomes of two murid rodents, *Mus caroli* and *Mus pahari*. They chose these two relatives of the well-studied laboratory mouse (*Mus musculus*) because previous work had shown that *M. caroli* and *M. pahari* shared a common ancestor with *M. musculus* approximately 3 million years ago and 6 million years ago, respectively. Together with the rat (*Rattus norvegicus*), this constitutes a clade of four rodents with high-quality genome sequences that Thybert et al. explicitly present as a parallel to the hominid clade of humans, chimpanzees, gorillas, and orangutans. Using the newly included murid genomes, Thybert et al. have addressed various questions regarding patterns of genomic change in these two mammalian groups. When all we had available were the human, chimpanzee, and mouse genomes, one could ask questions regarding differences in gene content or genomic organization between representative primates and a representative rodent. But with clades of genomes within each lineage, Thybert and colleagues have compared patterns of evolutionary change at a much higher resolution and have begun addressing the temporal dynamics of specific aspects of genome evolution that have not been previously investigated in these lineages.

It has long been known that rodents exhibit a higher rate of large (multi-megabase) chromosomal rearrangements than do primates. New intra-family comparisons show that there have been periods of rapid rearrangement interspersed with periods of much slower change during murid evolution. New assemblies also show that other features of the murid genome evolve more rapidly than they do in primates, including single nucleotide changes (previously reported, but now examined across multiple rodent species), SINE elements, CTCF-binding motifs, and, particularly, LINE elements. The evidence suggests that the expansion of LINE elements led to various changes in genome function in the genus *Mus*. In one case, new LINE elements specific to murid rodents accumulated within the androgen-binding protein gene cluster, apparently contributing to gene copy number increases in murids, particularly *M. musculus*. This gene family has been associated with behavioral mate choice and the dynamics of a hybrid zone between two mouse subspecies [3]. New analyses demonstrate that LINE element expansion in murid rodents differs significantly from that in the parallel clade of hominids, which has interesting biological consequences.

Another example of the value of more extensive sampling of genomes within and across clades is the novel finding by Thybert and colleagues that, although the B2 SINE element has increased in number in all three *Mus* species, only *M. caroli* displays a novel CTCF-binding site within the B2 element. Consequently, only *M. caroli* has increased CTCF-binding site numbers due to SINE element proliferation. Given the potential functional impact of increased CTCF-binding sites, this has considerable implications for understanding the evolution of gene regulation across rodent lineages. Overall, Thybert et al. observed that murid genomes exhibit faster evolution than do primates across multiple domains (coding exons, introns, regulatory sequences, ancestral repeat elements, and even inter-chromosomal rearrangements), but that episodes of accelerated change can occur independently in different murid lineages and that some of these rates fluctuate over evolutionary timescales within a given lineage.

This analysis by Thybert et al. provides a welcome example of the insights to be gained by comparing multiple genomes across several major lineages (families, superfamilies, or orders among mammals, or equivalent phylogenetic diversity in other taxonomic groups). A 2014 analysis of 48 bird genomes was another clear illustration of the value of this approach [4]. Other recent studies of smaller data sets have also indicated how comparisons made locally within a clade, when performed in parallel with comparisons across widely divergent lineages, can generate novel unanticipated results [5, 6].

## No longer just dreams

Several projects are now underway that promise to dramatically increase the number of species for which high-quality genome assemblies are available. Additional assemblies for a wider range of primates are in progress or have recently been published [7]. This is incrementally generating new clades of assemblies on additional branches of the primate evolutionary tree. At a considerably broader scale, the Vertebrate Genomes Project (VGP) is an international, multi-institution program designed to achieve impressive goals starting with the assembly of the genome of one species from each of approximately 260 vertebrate orders [8]. The VGP has established explicit recommended standards for the quality of new reference assemblies and plans to sequence many more species in its second and subsequent phases, reaching tens of thousands of new high-quality assemblies over several years. The insect i5k project is another such effort that intends to produce de novo assemblies for 1000 arthropods [9].

The continuing advances in genome analysis technology, involving methods for both data generation and computational analysis, are making truly revolutionary sequencing projects feasible. One can foresee a not-too-distant future in which almost gapless high-accuracy whole-genome assemblies are publicly available for tens of thousands of plant, invertebrate, and vertebrate species. Goals that were inconceivable even a few years ago are now under serious discussion [10]. A golden age of comparative genomics is coming. The recent paper by Thybert et al. [2] takes a welcome step towards the day when biologists will have ready access to extensive genomic information for entire clades of species.
